# The primacy of species turnover over intraspecific variation in the environmental filtering of understory ferns

**DOI:** 10.3389/fpls.2026.1779523

**Published:** 2026-02-25

**Authors:** Yuhan Zhou, Zhenzhen Zhang, Heming Liu, Shan Jiang, Zemei Zheng, Guochun Shen, Xihua Wang, Qingsong Yang

**Affiliations:** 1 Zhejiang Tiantong Forest Ecosystem National Observation and Research Station, School of Ecological and Environmental Sciences, East China Normal University, Shanghai, China; 2 Eastern China Conservation Centre for Wild Endangered Plant Resources, Shanghai Chenshan Botanical Garden, Shanghai, China

**Keywords:** community functional composition, community-weighted mean (CWM), environmental filtering, forest understory, intraspecific trait variation (ITV), species turnover

## Abstract

**Introduction:**

Quantifying community-level trait shifts, driven by species turnover and intraspecific trait variation (ITV), is essential for understanding environmental filtering and elucidating community assembly and species coexistence. While well-studied in seed plants, the relative roles of these processes in ferns—a key component of forest understories—remain poorly understood.

**Methods:**

Here, we evaluated how topographic, soil, and overstory biotic factors influence the functional traits of understory fern communities at a local scale in a subtropical forest. We measured six key functional traits across 45 fern species in 121 plots of 10 m × 10 m.

**Results:**

We found that trait-environment models based on species turnover alone (CWM_fixed) had consistently higher explanatory power than models that included ITV (CWM_specific) (mean pseudo-R² = 0.56 vs. 0.23). Variance partitioning revealed that trait-environment relationships were primarily driven by the unique effects of environmental factors rather than their shared variance, identifying soil properties and overstory biotic structure as distinct, independent drivers of community functional composition (explaining 23.0% and 17.7% of variance for plant growth and resource-use strategies, respectively).

**Discussion:**

Our results highlight two key insights: (1) the understory fern community responds to environmental filters primarily through species turnover (compositional shifts) rather than widespread intraspecific trait variation; (2) soil phosphorus and forest structure act as critical filters that together shape community-level functional traits of ferns.

## 1 Introduction

Plant functional traits, which reflect environmental selection and plant adaptive evolution (Cornelissen et al., 2003; Violle et al., 2007; Lajoie and Vellend, 2018), are pivotal in determining the ecological responses of plants to both biotic and abiotic environments (Shipley et al., 2016), and are key to explaining species coexistence and community assembly (Adler et al., 2013; Funk et al., 2017). Community-level shifts in functional traits arise from variation both among species (interspecific) and within species (intraspecific) (Bolnick et al., 2011). Interspecific trait variation reflects the screening effect of environmental filtering on community species composition, including species replacement or shifts in abundance along environmental gradients (McGill et al., 2006). Intraspecific trait variation reflects the phenotypic plasticity of species and is influenced by the extent of gene flow and the degree of environmental heterogeneity (Bolnick et al., 2003; Jung et al., 2010; Baythavong, 2011). Over recent decades, numerous studies have documented the patterns of plant trait variation at the community level along environmental gradients such as altitude, latitude and disturbance (Midolo et al., 2019; Ratier Backes et al., 2023; Ferrara et al., 2024). However, this body of research has been predominantly focused on seed plants (trees and shrubs) (Ratier Backes et al., 2023), leaving the functional ecology of ferns, the second-largest group of vascular plants, comparatively under-explored.

Ferns possess several unique biological characteristics that distinguish them from seed plants and justify their separate investigation (Smith, 1972; Christenhusz and Byng, 2016). Their reproduction via wind- or water-dispersed spores is independent of animal pollinators (Mehltreter et al., 2010), and their common rhizomatous growth form differs fundamentally from the arborescent architecture of most trees. Critically, ferns exhibit remarkable evolutionary conservatism, with fossil evidence indicating a much slower rate of morphological change compared to seed plants (Schneider et al., 2004; Bomfleur et al., 2014). Such long-term morphological stasis suggests a corresponding stability in physiological and ecological requirements—a phenomenon indicative of phylogenetic niche conservatism (Losos, 2008). Specifically, we hypothesize that the deeply conserved structural traits of ferns may impose physiological constraints, potentially limiting the ability of lineages to expand beyond their ancestral environmental niches (Wiens and Graham, 2005). Consistent with this hypothesis, ferns have been shown to exhibit phylogenetic conservatism associated with critical edaphic conditions, such as soil fertility (Lehtonen et al., 2015; Hernandez-Rojas et al., 2021; Nascimento da Costa et al., 2023). This phylogenetic inertia suggests that community responses to environmental gradients may be driven primarily by species replacement rather than intraspecific adaptive adjustments.

As highly sensitive ecological indicators, ferns are strongly influenced by local environmental conditions (Della, 2022). At fine spatial scales, their distribution and traits patterns are non-random, reflecting variations in soil properties (e.g., texture and fertility), topography, humidity, and light availability (Tuomisto and Poulsen, 2000; Nóbrega et al., 2011; Patil et al., 2016; Della and Falkenberg, 2019; Viana et al., 2021; Landeros-López et al., 2025). For instance, leaf area often decreases in drier habitats (Cicuzza et al., 2024), while communities on low-fertility soils may exhibit higher leaf dry matter content and lower specific leaf area—traits associated with a resource-conservative strategy (Kessler et al., 2007; Viana and Dalling, 2022). While these patterns are increasingly recognized, a comprehensive understanding of how multiple environmental factors jointly shape fern community traits, and the relative roles of interspecific versus intraspecific variation in driving these relationships, remains elusive.

This knowledge gap is particularly salient in the evergreen broad-leaved forests (EBLFs) of subtropical China, where ferns are a dominant component of the understory (Wang et al., 2007; Liu et al., 2022). While trait-environment relationships for canopy trees in these forests are relatively well-studied (e.g., Liu et al., 2012; He et al., 2018; Li et al., 2022), the understory fern community has received scant attention. The understory presents a distinct set of conditions, characterized by limited light and a more stable microclimate, to which ferns are particularly adapted due to traits like passive stomatal control and lower hydraulic conductivity (Watkins et al., 2010; Brodribb and McAdam, 2011). Consequently, extrapolating ecological strategies from trees to ferns is problematic, warranting a dedicated investigation.

In this study, we investigated six key functional traits related to plant growth and resource-use strategies of understory ferns across 121 plots in a subtropical EBLF in eastern China. We sought to determine how topographic, soil, and overstory biotic factors influence the functional composition of understory fern communities at a local scale, while explicitly accounting for potential spatial autocorrelation. Our primary objectives were to: (1) assess whether incorporating intraspecific trait variation (ITV) improves the explanatory power of trait-environment models; and (2) evaluate how topography, soil properties, and biotic factors (tree layer structure and composition) independently and jointly influence community-weighted mean (CWM) traits.

## 2 Materials and methods

### 2.1 Study area and design

**Figure 1:**
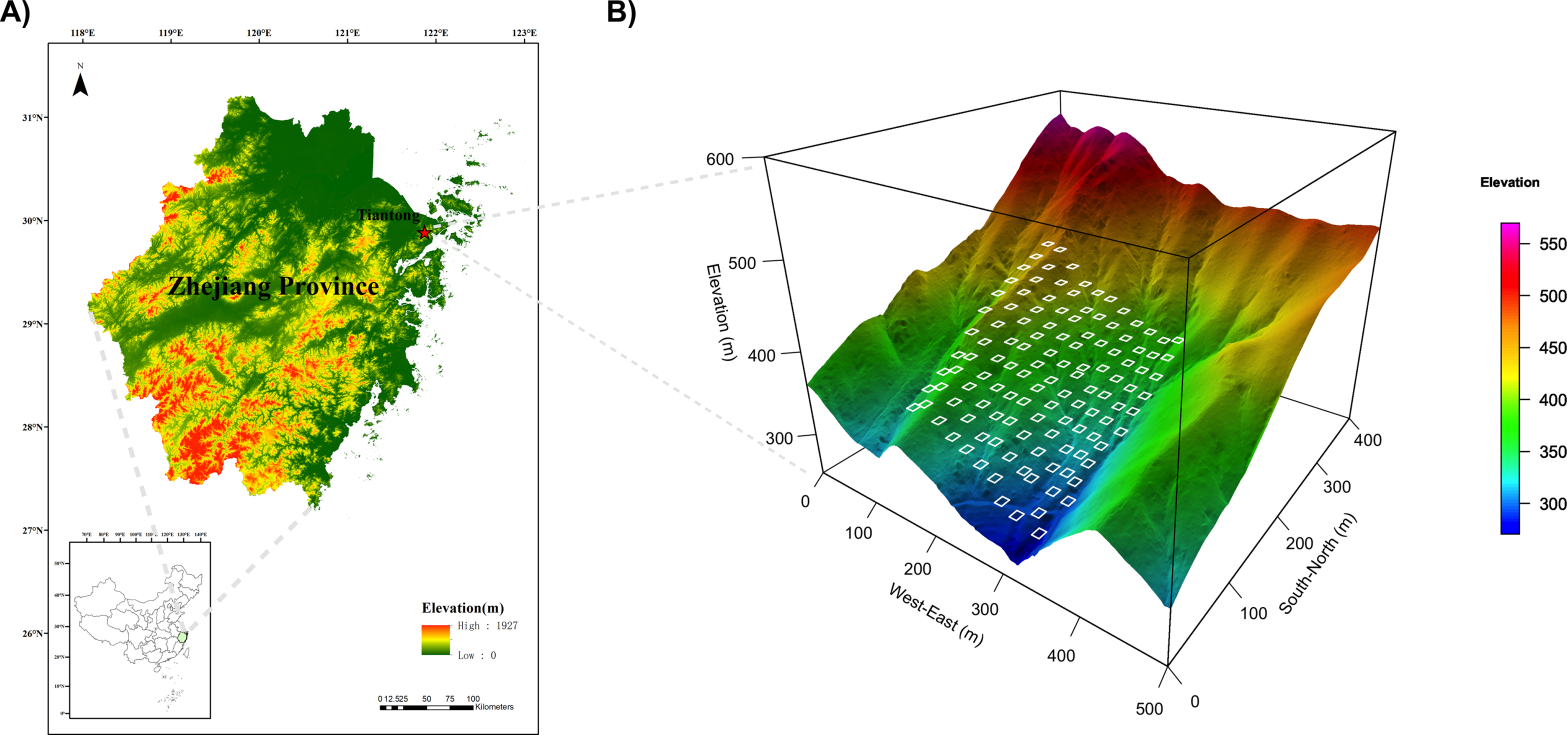
Map of the study area. **(A)** Location of the study site in Zhejiang Province, China. **(B)** Topographic map showing the spatial distribution of the 121 sampling quadrats within the permanent forest plot.

The study site is situated within Tiantong National Forest Park (29^°^48′N, 121^°^47′E) in Zhejiang Province, eastern China (**Figure 1A**). This region experiences a typical subtropical monsoon climate, characterized by warm, moist summers and dry, cold winters (Wang et al., 2007). The mean annual temperature is 16.2 ^°^C; the warmest and coldest months are July and January, with a mean temperature of 28.1 ^°^C and 4.2 ^°^C, respectively. This region supports EBLF, primarily dominated by species belonging to the Fagaceae and Theaceae families (Ren et al., 2021).

This study was conducted in a 4.84-ha undisturbed core area (220 m × 220 m) of a 20-ha permanent forest plot established following the ForestGEO protocol (Condit, 1998; Yang et al., 2016). All free-standing trees with a diameter at breast height ≥ 1 cm were tagged, mapped, measured, and identified to species every five years. For this study, a total of 121 quadrats (10 m × 10 m) were uniformly selected to survey and collect tree and understory data (**Figure 1B**). The woody plant community is notably dominated by species such as *Eurya loquaiana* and *Litsea elongata*; detailed information on Importance Values (IV) and leaf habits is provided in **Supplementary Table 1**.

A total of 45 fern species were recorded in the summers of 2024 and 2025 (**Supplementary Table 2**). The cover of herbaceous plants in each quadrat ranges from 4% to 95%, and the cover of ferns ranges from 3% to 83%. Ferns were the dominant component of the herbaceous layer, accounting for an average of 69.6% of the total cover in each plot. The distributions of relative fern cover and species richness across the study plots are illustrated in **Supplementary Figure 1**. The dominance ranking of community, listing the top 20 species ranked by Importance Value (IV), is provided in **Supplementary Figure 2**.

### 2.2 Understory fern functional traits

Six functional traits were measured: fern height (FH), leaf area (LA), specific leaf area (SLA), leaf dry matter content (LDMC), chlorophyll content (SPAD), and actual quantum yield of photosystem II (Φ_PSII_). FH serves as an indicator of plant performance (Violle et al., 2007), and variations in height among individuals may reflect asymmetric competition for light (Blondeel et al., 2020; Herben and Austin, 2016). Smaller LA is generally associated with reduced water loss and enhanced tolerance to nutritional stress (Niinemets et al., 2007). SLA, defined as the ratio of total leaf area to total leaf dry mass, reflects a trade-off between mass-based photosynthetic capacity and leaf life span (Wright et al., 2004). LDMC, calculated as the ratio of leaf dry mass to fresh mass, is positively correlated with leaf lifespan and the plant’s ability for nutrient retention (Wright et al., 2004). SPAD plays a vital role in absorbing, transferring, and converting light energy during photosynthesis. Its content and composition are influenced by the light environment (Boardman, 1977; Croft et al., 2017). Φ_PSII_ quantifies the efficiency of light energy use by the photosystems II (Maxwell and Johnson, 2000).

Traits were measured on mature and healthy fronds from five randomly selected, spatially distinct individuals (or ramets) per fern species in each plot. All trait measurements followed the standard protocols of Pérez-Harguindeguy et al. (2013); for species with dimorphic fronds, all measurements were taken exclusively on the sterile fronds. In total, 2273 samples from 45 fern species were collected to measure their leaf functional traits. FH and SPAD were measured in the field. FH was measured as the vertical distance from the plant base to the apex of the youngest fully expanded leaf, without stretching the axis. SPAD was recorded on fully expanded leaves using a SPAD-502 Plus chlorophyll meter (Konica-Minolta, Japan), with three measurements per leaf averaged (Marenco et al., 2009). In the laboratory, fresh mass and leaf area were determined within 12 hours using an analytical balance and a Li-Cor Portable Area Meter Li-3000 (Li-Cor Biosciences, Nebraska, USA). Φ_PSII_ was assessed using a highly portable modulated chlorophyll fluorometer (MINI-PAM-II) after 30 minutes of dark adaptation. Leaves were then dried at 75°C for 72 h to obtain leaf dry mass. SLA and LDMC were calculated following standard protocols.

**Figure 2:**
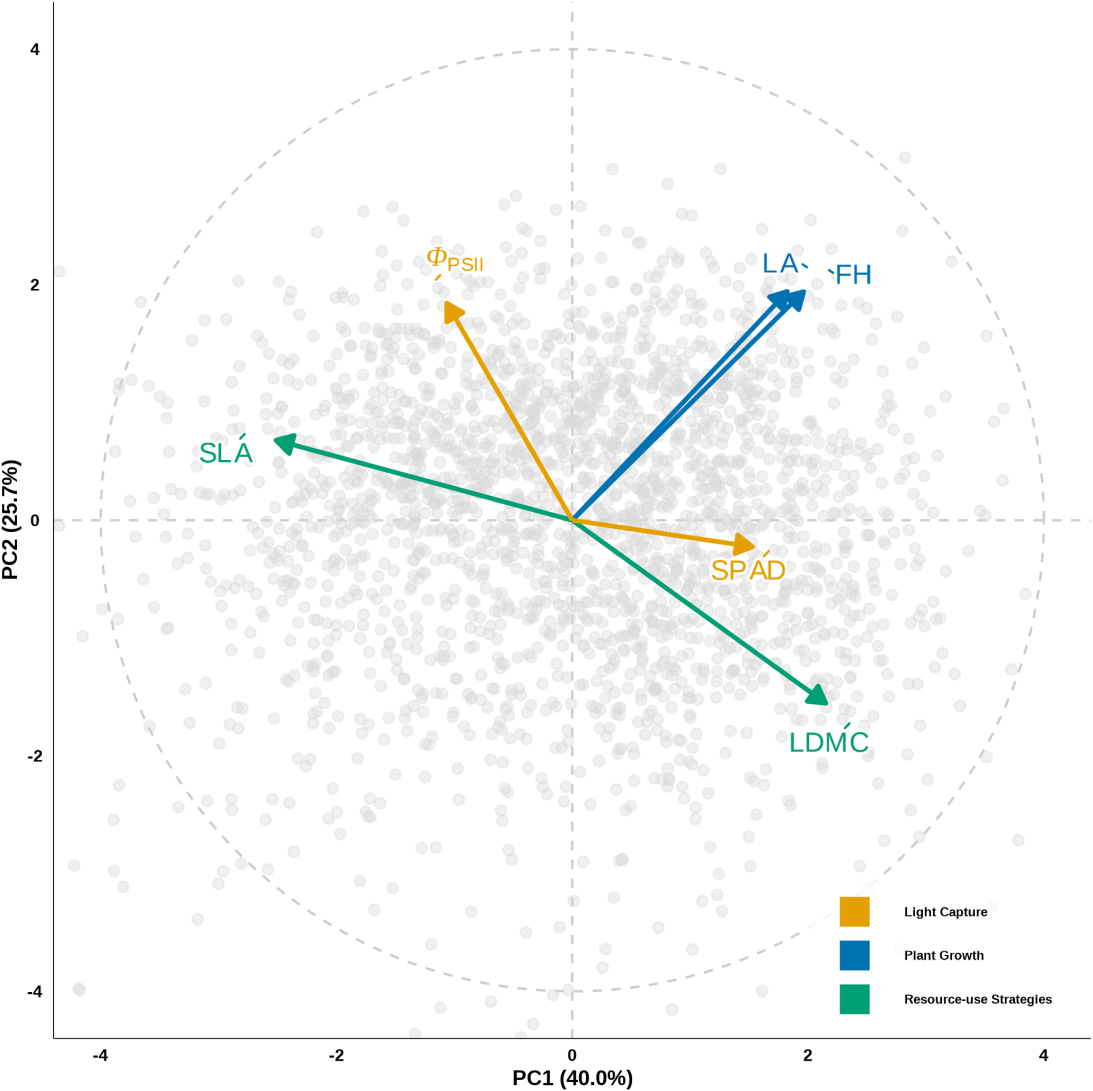
Principal component analysis (PCA) of functional traits for the understory fern species. The biplot illustrates the correlations among the six functional traits and defines the multidimensional functional space. The first axis (PC1) represents a spectrum of ‘Resource-use Strategies’ (primarily loaded by SLA and LDMC), while the second axis (PC2) represents ‘Plant Growth’ (primarily loaded by FH and LA). The arrows indicate the direction and relative loading of each trait on the principal axes (scaled to a circle with radius 4 to improve visibility).

Given that plant functional traits commonly covary due to underlying physiological and evolutionary constraints, a Principal Component Analysis (PCA) was performed on the trait dataset to extract the primary axes of functional variation and reduce dimensionality. The first two principal components (PCs) cumulatively explained 65.7% of the total variation (**Figure 2**). The first axis (PC1, explaining 40.0% of the variance) was defined as ‘Resource-use Strategies’, representing a gradient from acquisitive to conservative strategies, and was negatively correlated with SLA and positively correlated with LDMC. The second axis (PC2, explaining 25.7% of the variance) represented ‘Plant Growth’, driven mainly by positive loadings for FH and LA.

### 2.3 Abiotic factors

Abiotic factors were quantified from four topographic and nine soil properties (**Table 1**). Topographic variables (elevation, slope, aspect and convexity) were calculated for each 10 m × 10 m quadrat (Yang et al., 2011). Soil samples were collected and analyzed for pH, water content, total nitrogen (TN), soil organic carbon (SOC), total phosphorus (TP), potassium (K), calcium (Ca), magnesium (Mg), and manganese (Mn) following John et al. (2007). Point measurements of soil properties were spatially interpolated to all quadrats using standard block kriging (Goovaerts, 1997; John et al., 2007).

**Table 1:** Overview of abiotic and biotic predictor variables used to explain community-weighted mean and variation in functional traits of fern.

Variable group	Variable name	Description	Mean ± SD (Range)	Unit
Topographic	Elevation	Mean elevation of the four corners of each quadrat	400.28 **±** 39.18 (318.54, 490.23)	m
	Slope	Mean slope of the quadrat	32.48 ± 5.10 (18.54, 41.77)	°
	Aspect	Direction of the slope face	152.21 **±** 30.09 (101.54, 230.67)	°
	Convexity	Elevation of focal quadrat minus the mean elevation of eight surrounding quadrats (or four corners at edge)	0.02 **±** 0.85 (-2.03, 2.67)	m
Soil	pH	Soil acidity measured from topsoil (0–10 cm)	3.79 **±** 0.21 (3.31, 4.31)	/
	Soil Moisture	Gravimetric soil moisture	35.42 **±** 4.13 (28.62, 49.85)	%
	Soil organic carbon (SOC)	Total concentration of organic carbon (C) in topsoil	7.88 ± 2.58 (4.95, 18.05)	%
	Total nitrogen (TN)	Total concentration of nitrogen (N) in topsoil	0.50 ± 0.12 (0.35, 0.92)	%
	Total phosphorus (TP)	Total concentration of phosphorus (P) in topsoil	0.03 ± 0.007 (0.02, 0.05)	%
	Ca	Calcium in topsoil	1900 ± 500 (1200, 3100)	mg/kg
	K	Potassium in topsoil	22700 ± 3600 (13000, 29900)	mg/kg
	Mg	Magnesium in topsoil	4100 ± 600 (2600, 5500)	mg/kg
	Mn	Manganese in topsoil	548.29 ± 271.42 (144.01, 1323.65)	ppm
Biotic	Density	Number of individual trees per 10 m × 10 m quadrat (i.e., Stand density)	67 ± 30 (0, 182)	ind/100 m²
	Basal area	Total basal area of trees per 10 m × 10 m quadrat	35.52 ± 18.02 (0, 87.02)	m ^2^/ha
	NMDS1	First axis of NMDS ordination (community composition)	0 ± 0.49 (-1.22, 1.11)	/
	NMDS2	Second axis of NMDS ordination (community composition)	0 ± 0.32 (-1.2, 0.57)	/

**Figure 3:**
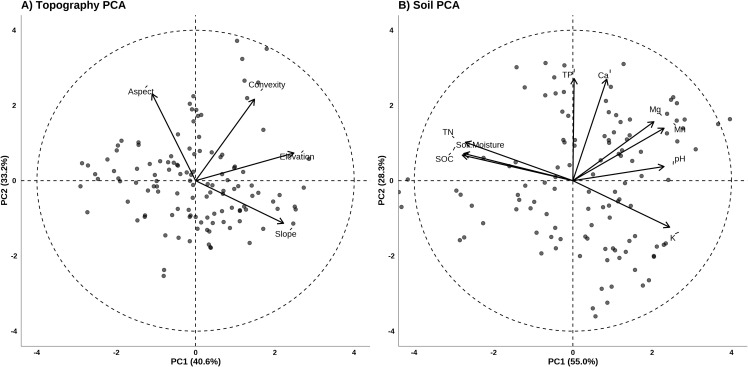
Principal components analysis (PCA) plots for **(A)** topography, **(B)** soil. The arrows indicate the relative loadings of each explanatory variable (scaled to a circle with radius 4 to improve visibility).

To reduce dimensionality and multicollinearity, independent Principal Component Analyses (PCA) were performed for the topographic (4 variables) and soil (9 variables) datasets. The first two principal components (PCs) from the topography and soil PCAs were retained for subsequent analyses; these collectively accounted for 73.8% and 83.3% of the total variance in their respective datasets (**Figure 3**).

The first topographic PC axis (PC_Topo-ele_) was primarily positively correlated with elevation, while the second axis (PC_Topo-asp_) was associated with aspect (**Figure 3A**). The first soil PC axis (PC_Soil-pH_) correlated positively with pH and negatively with TN, SOC, and soil moisture. The second soil PC axis (PC_Soil-P_) was primarily driven by TP and Ca content (**Figure 3B**).

### 2.4 Biotic factors

The overstory biotic environment was characterized using four variables: tree stand density, basal area, and the first two axes of a non-metric multidimensional scaling (NMDS) ordination of tree species composition (NMDS1 and NMDS2; **Table 1**). Stand density was calculated as the number of individual trees (DBH ≥ 1 cm) per quadrat. Total basal area was the sum of the cross-sectional stem areas at breast height for all trees in a quadrat (Condit, 1998; Forrester and Bauhus, 2016). An NMDS ordination was performed based on a matrix of species Importance Values (IV) using the “metaMDS” and “MDSRotate” functions in vegan package in R (Fortin and Dale, 2005; Oksanen et al., 2024). The first two NMDS axes (NMDS1 and NMDS2), representing the primary gradients in community composition, were extracted for analysis (**Supplementary Figure 3**). NMDS1 captured a gradient from simple, low-stature communities to complex, multi-layered canopies in moist habitats. NMDS2 primarily reflected a shift from evergreen to deciduous species, indicating changes in the light environment beneath the forest canopy.

### 2.5 Statistical analysis

We calculated three types of community-weighted mean (CWM) for each trait and the two PCA axes in each plot, weighted by the relative cover of fern species (Garnier et al., 2004; Wieczynski et al., 2019; Garbowski et al., 2024).

CWM_fixed_ was calculated based on a fixed mean trait value per species averaged across all plots, reflecting only species turnover effects (Equation 1).

(1) $$CWM_{fixed}=\sum_{i=1}^{s}p_{i}x_{i}$$

where pi is the weight of the i-th species (cover), *S* is the number of species, and *x_i_* is the fixed mean trait value (or PC score) of the i-th species averaged across all plots where the species is found.

CWM_specific_ was calculated using plot-specific mean trait values, incorporating both ITV and species turnover (Equation 2).

(2) $$CWM_{specific}=\sum_{i=1}^{s}p_{i}x_{im}$$

where *x_im_* is the specific mean trait value (or PC score) of the i-th species, which is valid just for a given plot sampled.

CWM_intravar_, representing the community-level trait variation attributable solely to ITV, was derived as the difference: CWM_intravar_ = CWM_specific_ − CWM_fixed_ (Siefert et al., 2015). In this equation, the sign of CWM_intravar_ (positive or negative) indicates the direction of the community’s trait shift away from the fixed species mean, while its magnitude reflects the strength of the ITV effect (**Table 2**).

**Table 2:** Summary statistics of the six functional traits and their community-weighted means (CWM) across 121 plots.

Trait (unit)	CWM_fixed_	CWM_specific_	CWM_intravar_
FH (cm)	42.69 (28.25, 54.00)	42.83 (26.00, 98.70)	0.14 (-20.27, 50.67)
LA (cm^2^)	437.16 (190.57, 812.18)	442.98 (167.79, 1324.87)	5.82 (-328.19, 626.69)
SLA (cm^2^/g)	196.95 (154.45, 300.83)	193.71 (119.29, 302.59)	-3.24 (-68.14, 106.55)
LDMC	0.37 (0.25, 0.50)	0.37 (0.22, 0.57)	0 (-0.12, 0.13)
SPAD	43.97 (33.68, 49.15)	43.94 (30.94, 55.51)	-0.03 (-12.59, 7.33)
Φ_PSII_	0.32 (0.25, 0.38)	0.32 (0.14, 0.43)	0 (-0.13, 0.12)

Values indicate the mean (range). CWM, community-weighted mean, fixed: based on a single mean per species (turnover), specific: accounted for intraspecific variation in plant traits (turnover + ITV), intravar: CWM_specific_ – CWM_fixed_: changes in CWM solely due to ITV.

To partition the sources of community-level trait variation, the relative contribution of ITV and species turnover was quantified following the sum of squares decomposition method (Lepš et al., 2011). This method decomposes the total sum of squares of the CWM_specific_ values (SS_specific_) which represents the total community-level trait variation, into three components: the sum of squares explained by species turnover (SS_fixed_), the sum of squares explained purely by intraspecific trait variation (SS_intraspecific_) and the sum of squares explained by the covariation between them (SS_cov_), so that SS_specific_ = SS_fixed_ + SS_intraspecific_ + SS_cov_. We calculated the percentage contribution of each component to the total explained variation and visualized the results using stacked bar plots. We performed this decomposition for each of the six functional traits.

We used Generalized Least Squares (GLS) models to evaluate the effect of eight environmental predictors (PC_Topo-ele_, PC_Topo-asp_, PC_Soil-pH_, PC_Soil-P_, stand density, total basal area, NMDS1, and NMDS2) on community functional composition while accounting for spatial autocorrelation. Separate models were fitted for each of the two functional axes (PC1 and PC2) and the six traits, using CWM_specific_, CWM_fixed_, and CWM_intravar_ as response variables. All eight predictors were standardized to interpret their relative importance on a comparable scale (Gross et al., 2017). Potential multicollinearity was assessed by calculating variance inflation factors (VIF), and all VIF values were found to be below 5 (**Supplementary Table 3**). The overall explanatory power of each model was assessed using Nagelkerke’s pseudo-R-squared.

To further quantify the independent and shared effects of these environmental factor groups (topographic, soil, and biotic), we performed a variance partitioning analysis based on the Nagelkerke’s pseudo-R^2^ values derived from the GLS models (Pinheiro and Bates, 2000; Pinheiro et al., 2024). All statistical analyses were conducted in R (version 4.4.1, R Core Team, 2024). Analyses relied on several packages, including nlme, tidyverse, FactoMineR, vegan, ggplot2 and eulerr. Data exploration, model evaluation, and graphical visualization closely followed the comprehensive framework proposed by Govaert et al. (2024), which provides a robust standard for analyzing trait-environment relationships. We adapted their R code workflow to specifically address the ecological context of understory fern communities.

## 3 Results

### 3.1 Relative roles of species turnover and intraspecific trait variation

**Figure 4:**
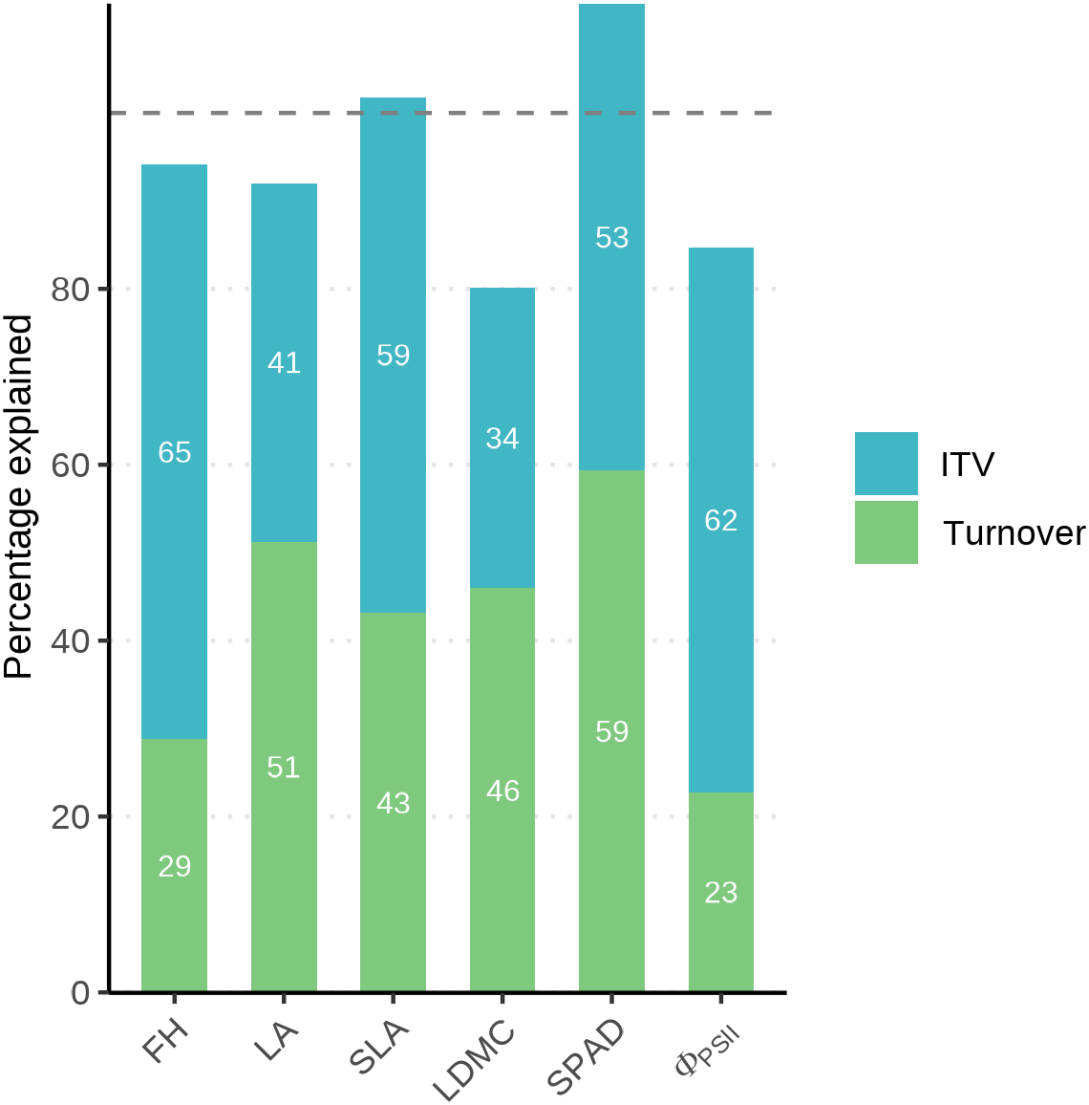
Relative contribution of species turnover and intraspecific trait variation (ITV) to the total community-level trait variation of community functional traits. The total variance was partitioned into species turnover (green bars), ITV (blue bars), and their covariation. Note that for some traits (e.g., SLA and SPAD), the sum of turnover and ITV contributions exceeds 100%. This indicates a negative covariation between the two components, implying that shifts in species composition and intraspecific variability respond to environmental gradients in opposite directions, thereby partially offsetting each other at the community level.

The total variance in CWM traits was partitioned into components of species turnover and ITV (**Figure 4**). The contribution of species turnover ranged from 23% to 59%, while that of ITV ranged from 34% to 65%. Species turnover made a greater contribution to the total variance of LA, LDMC, and SPAD, whereas ITV accounted for a larger proportion of variance in FH, SLA, and Φ_PSII_. Notably, we observed a negative covariation between species turnover and ITV for SLA and SPAD, resulting in the sum of the independent contributions of species turnover and ITV exceeding 100% of the total community-level trait variance.

**Figure 5:**
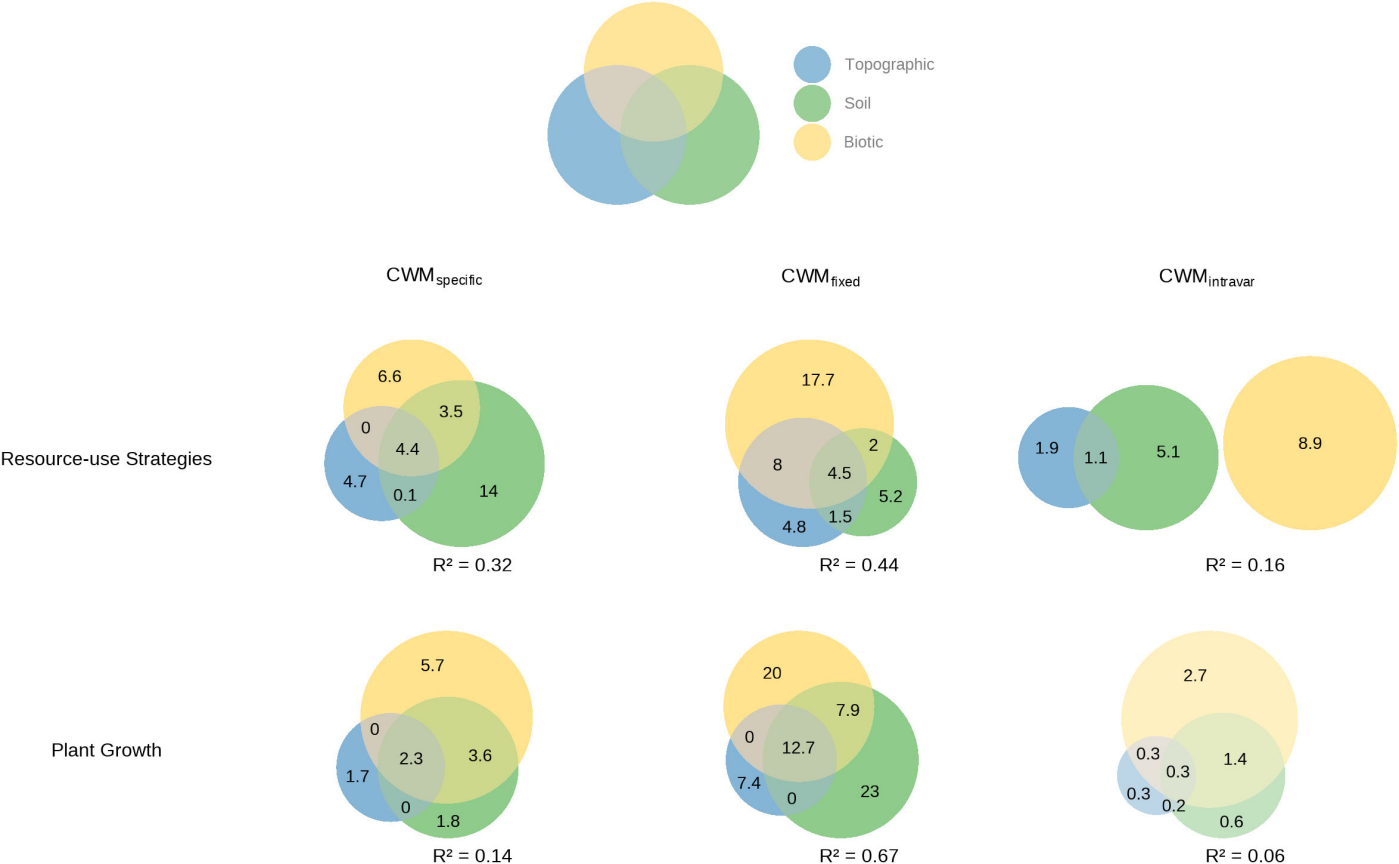
Relative contributions of topographic, soil, and biotic factors to the community functional composition (PC1 and PC2) of understory ferns. The figure shows the results from the variance partitioning analysis based on Generalized Least Squares (GLS) models accounting for spatial autocorrelation. The values in each Venn diagram represent the percentage of variation (Nagelkerke’s pseudo-R^2^) explained by each component for the first two principal components: PC1 (‘Resource-use Strategies’) and PC2 (‘Plant Growth’). The non-overlapping areas of the ellipses represent the unique effects of a single factor group, while the overlapping areas represent the shared effects among factor groups. The total pseudo-R^2^ value noted below each diagram indicates the total variation explained by all three environmental factor groups combined.

Regarding environmental explanatory power, the GLS models revealed distinct drivers for functional community composition. A consistent pattern emerged across both the integrated functional axes (**Figure 5**) and the six individual traits (**Supplementary Figure 4**): models based on species turnover alone (CWM_fixed_) exhibited consistently higher explanatory power than models including ITV (CWM_specific_). For the integrated functional axes, the environmental models explained 32% of the variation for ‘Resource-use Strategies’ (increasing to 44% when considering turnover alone), and 14% for ‘Plant Growth’ (vs. 67% for turnover). Models for CWM_intravar_ (ITV alone) showed limited environmental explanatory power across integrated functional axes (16% and 6%). For the individual traits, the reduction in explanatory power when including ITV was most evident for SPAD (pseudo-R^2^ decreased from 0.32 to 0.11) (**Supplementary Figure 4**). Models for CWM_intravar_ (ITV alone) showed limited environmental explanatory power across all traits (pseudo-R^2^ < 0.11).

### 3.2 Relative importance of environmental factor groups

Variance partitioning revealed that topographic, soil, and biotic factors influenced the CWM of plant functional traits through both their unique effects and shared effects (**Figure 5**). For CWM_fixed_ models, the primary environmental drivers differed markedly between the two axes. The unique effect of biotic factors was the strongest explanatory component for ‘Resource-use Strategies’ (17.7%), while the unique effect of soil factors was dominant for ‘Plant Growth’ (23.0%), followed closely by the unique effect of biotic factors (20.0%). For CWM_specific_ models, the relative importance of these drivers shifted. The unique effect of soil factors became the largest contributor for ‘Resource-use Strategies’ (14.0%), whereas for ‘Plant Growth’, the unique effect of biotic factors explained the largest proportion of variance (5.7%).

Regarding individual traits, the relative importance of environmental drivers varied considerably (**Supplementary Figure 4**). For CWM_fixed_ models, a clear divergence was observed between trait types: biotic factors were the primary drivers for leaf physiological traits, explaining the largest proportion of variation for SPAD (21.7%) and SLA (18.5%). In contrast, soil factors played a leading role for morphological traits, particularly for LA (13.4%) and FH (11.1%). For CWM_specific_ models, the dominant drivers shifted for certain traits. Notably, the primary driver for SLA shifted from biotic to soil factors (7.9%), while the driver for FH shifted from soil to biotic factors (9.4%). Finally, for LDMC and Φ_PSII_, the effects of all three environmental factor groups were negligible (R^2^≈0) in both model types.

### 3.3 Trait-environment relationships

**Figure 6:**
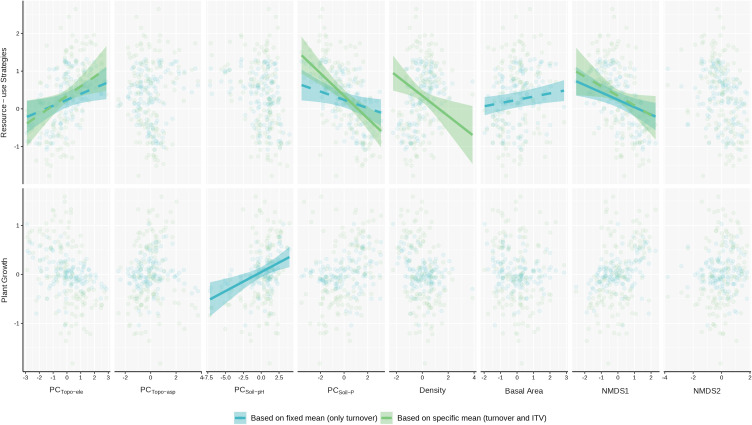
Unique effects of significant environmental predictors on the integrated functional axes (PC1 and PC2) of understory fern communities. Each panel displays the predicted relationship between a functional axis (PC1: ‘Resource-use Strategies’ or PC2: ‘Plant Growth’) and a single environmental predictor variable. Relationships are derived from Generalized Least Squares (GLS) models that account for spatial autocorrelation while statistically controlling for the effects of other predictors (holding them at their mean values). Models based on CWM_specific_ are shown in green, and those based on CWM_fixed_ are in blue-green. Trend lines are shown for significant predictors, with line style indicating the level of significance: solid for *p* < 0.01 and dashed for *p* < 0.05. Non-significant relationships (*p* ≥ 0.05) are not displayed.

Regarding abiotic drivers, soil factors were the primary predictors for both integrated functional axes (**Figure 6**) and individual traits (**Supplementary Figure 5**). PC_Soil-P_ (positively associated with soil phosphorus and calcium) was negatively associated with the ‘Resource-use Strategies’ axis across both the CWM_fixed_ and CWM_specific_ models, while PC_Soil-pH_ was positively associated with the ‘Plant Growth’ axis in the CWM_fixed_ model. Reflecting these broad patterns at the individual trait level, PC_Soil-P_ exhibited the most widespread effects: increasing values were associated with increased SLA, SPAD, and Φ_PSII_, and decreased FH, LA, and LDMC. These relationships were generally consistent between the CWM_fixed_ and CWM_specific_ models, although the positive association with SLA was significant only in the CWM_specific_ model, and some associations weakened or became non-significant (e.g., SPAD) when ITV was included. Regarding topographic factors, PC_Topo-ele_ showed a significant positive correlation with ‘Resource-use Strategies’ axis across both models. In contrast, PC_Topo-asp_ primarily influenced individual morphological traits, showing associations with increases in FH and LA and a decrease in SPAD in the CWM_fixed_ model.

Biotic factors also significantly influenced the functional axes and fern traits (**Figure 6**; **Supplementary Figure 5**). Stand density showed a consistent negative correlation with the ‘Resource-use Strategies’ axis in the CWM_specific_ model, whereas total basal area was positively associated with this axis in the CWM_fixed_ model. NMDS1 exerted a consistently negative influence on the ‘Resource-use Strategies’ axis across both the CWM_fixed_ and CWM_specific_ models. At the individual trait level, stand density exhibited consistent negative correlations with FH and LA across both model types. SPAD and Φ_PSII_ were significantly correlated with stand density only in the CWM_fixed_ model, while LDMC showed a significant response only in the CWM_specific_ model. Total basal area influenced traits exclusively in the CWM_fixed_ model (positive with LA, negative with Φ_PSII_). NMDS1 was strongly correlated with four traits in the CWM_fixed_ model (negatively with LA and LDMC; positively with SLA and Φ_PSII_). In the CWM_specific_ model, only the negative correlation with LDMC remained significant. NMDS2 only showed consistent positive relationships with photosynthetic traits (SPAD and Φ_PSII_) in CWM_fixed_ models.

## 4 Discussion

Our study reveals that functional variation in the understory fern community is primarily driven by species turnover along local environmental gradients, with intraspecific trait variation (ITV) playing a substantial but environmentally idiosyncratic role. This turnover-dominated response is characterized by the strong independent filtering effects of soil and biotic factors, highlights niche-based processes and the distinct roles of different environmental factor groups in shaping this ancient plant lineage’s community assembly.

### 4.1 The role of intraspecific trait variation

We found that while ITV accounted for a significant proportion (34–65%) of total fern community trait variance (**Figure 4**) as some previous studies (Siefert et al., 2015; Wang et al., 2022), its inclusion in community-weighted means (CWM_specific_) consistently weakened the explanatory power of trait-environment models compared to models based on species mean traits alone (CWM_fixed_) (**Figure 5**; **Supplementary Figures 4**, **6**). This attenuation of model fit is directly attributable to the limited environmental predictability of the ITV component itself, as evidenced by the low pseudo-R^2^ values for CWM_intravar_ models across both integrated functional axes and individual traits (**Figure 5**; **Supplementary Figure 4**). This suggests that the high magnitude of ITV does not track environmental gradients in the same direction or strength as species turnover. Instead, it likely arises from species-specific (idiosyncratic) behaviors (Westerband et al., 2021; Albert et al., 2010). Our species-level analysis supports this: only a minority of species showed significant trait responses to any given gradient, and the few responding species were influenced by different environmental factors (**Supplementary Table 4**; **Supplementary Figure 7**). For example, the SLA of *Diplopterygium glaucum* responded significantly only to topography, while that of *Woodwardia japonica* responded to elevation and stand density (**Supplementary Figure 5**). Similarly, for single trait like LDMC, *Diplopterygium glaucum* showed no significant relationship with any measured environmental factors, whereas *Woodwardia japonica* responded to soil P (**Supplementary Figure 7**). This combination of prevalent non-responsiveness and divergent responses among the few responsive species results in a community-level ITV signal that is largely decoupled from the dominant environmental gradients, explaining its weak aggregate relationship.

This pattern suggests that community-level trait responses are primarily driven by shifts in species composition (turnover) rather than coordinated phenotypic adjustments. One potential interpretation for this dominance of turnover may lie in the hypothesis of evolutionary conservatism in ferns, where the rigid physiological constraints inherent in this ancient lineage may limit individual-level plasticity. This aligns perfectly with findings in other fern communities, where strong species turnover along elevation gradients is interpreted as direct evidence for this environmental determinism (Kessler et al., 2007; Merino et al., 2023). Previous research has further found that species turnover occurs significantly between closely related species, which suggests that the niche of a single species is relatively fixed, making it difficult to cross environmental gradients through self-adjustment and thus necessitating their replacement by pre-adapted related species (Lehnert, 2011; Lehnert et al., 2013; Lehnert, 2014). Alternatively, the high but idiosyncratic ITV we observed may simply reflect a scale mismatch, where individuals respond to fine-scale microenvironmental heterogeneity that was not captured at our 10×10 m plot scale (Albert et al., 2010). Finally, the observed pattern may also be interpreted as a stabilizing mechanism. The fact that the combined contributions of turnover and ITV frequently exceed 100% (**Figure 4**) reveals a negative covariation between these two components. This suggests that ITV acts as a functional buffer rather than a mere source of noise; as environmental shifts drive fern species replacement, idiosyncratic phenotypic adjustments within species may move in the opposite direction, thereby compensatory stabilizing the community’s overall functional structure (Lepš et al., 2011; Luo et al., 2016; Ferrara et al., 2024). Thus, the apparent independence of ITV from environmental gradients may represent an emergent property of a stabilizing mechanism that maintains functional continuity across varying environments.

### 4.2 Soil phosphorus as a key driver of community resource strategies

Soil gradients, particularly the phosphorus-richness gradient (PC_Soil-P_), emerged as a major driver of community functional traits. Along this gradient, we observed a clear, community-wide shift along the Leaf Economics Spectrum (Wright et al., 2004), from a resource-conservative to a resource-acquisitive strategy. This transition was captured by the ‘Resource-use Strategies’ axis (PC1) and manifested as a coordinated adjustment of traits, characterized by a significant increase in SLA and a concurrent decrease in LDMC (**Figures 2**, **6**). The fact that individual trait responses (SLA and LDMC) closely mirrored the shifts in the integrated PC1 axis further reflects the strong biological coupling within the fern leaf economics syndrome (**Supplementary Figure 5**). This suggests that understory ferns are not merely adjusting traits independently to track soil phosphorus, but are constrained by fundamental trade-offs that force a coordinated functional transition. These coordinated trait shifts strongly suggest that phosphorus acts as a primary limiting nutrient constraining the functional strategies of this fern community. This finding is supported by independent research, which identified this subtropical forest as “highly phosphorus-limited” based on soil respiration experiments (Liu et al., 2019). Our results demonstrate that this understory fern community responds to soil nutrient gradients with a sensitivity similar to that observed in trees (Jones et al., 2013), challenging the generalized view that understory herbs are primarily light-limited (Wang et al., 2022). It highlights the critical role of soil resource limitation even in light-poor understory environments.

### 4.3 Forest structure acts as a biotic filter

Biotic factors derived from the tree community exerted multiple influences on understory fern traits. Tree density showed the most consistent effects across models, indicating that microclimatic variation—primarily understory light availability—acts as a stable biotic filter shaping fern functional strategies (Sercu et al., 2017; Murakami et al., 2022; Jin et al., 2024). Specifically, ferns in denser stands exhibited smaller and thinner fronds, alongside higher SPAD values (**Figure 6**; **Supplementary Figure 5**). This conservative pattern is logical in a high-density understory, which creates a deep and persistent low-light environment. Our results show the fern community shifting towards a persistence-oriented strategy: it effectively “forgoes” costly vertical competition (smaller FH, LA) to minimize structural and respiratory costs, while simultaneously maximizing capture efficiency in the deep shade by increasing chlorophyll content (higher SPAD) (Anderson et al., 1995; Johnson et al., 2000; Rundel et al., 2020).

Total basal area, which reflects forest structural size and canopy biomass, affected several traits only in the CWM_fixed_ model. This suggests that as the forest matures, it imposes powerful, long-term structural filters on the understory, such as the accumulation of deep, recalcitrant litter layers and intense, persistent root competition from large, established trees (Çömez et al., 2021; Müller et al., 2021; Ou et al., 2015). These conditions filter for species pre-adapted to these substrate and microhabitat conditions, while excluding species that rely on different forest-floor environments. This explains why the relationship is visible only in the CWM_fixed_ model.

Tree species composition also served as a significant biotic filter. NMDS1, which captured a gradient from simple to complex, multi-layered canopies (**Supplementary Figure 3**), was associated with shifts in fern stature, leaf structure, and photosynthetic traits. NMDS2, reflecting a shift from evergreen to deciduous species, especially pioneer deciduous species like *Alangium kurzii* and *Clerodendrum cyrtophyllum*, showed consistent positive relationships with photosynthetic traits (SPAD and Φ_PSII_). The disappearance of most NMDS–trait relationships in the CWMspecific model further reinforces that these biotic influences operate predominantly through species turnover rather than ITV.

## 5 Conclusion

Our study provides a nuanced understanding of local-scale assembly in subtropical understory fern communities. The core finding is that species turnover is the dominant process linking fern community traits to environmental gradients. Including intraspecific trait variation does not strengthen but rather attenuates community-level trait-environment models, revealing a key difference in functional structuring compared to more plastic plant groups. Furthermore, the unique effects of soil and biotic factors were more important, highlighting that soil nutrients and canopy structure act as distinct, independent selective forces shaping the understory fern community.

These findings advance our understanding of fern functional ecology and underscore that the mechanisms of community assembly can be distinct even within the same forest ecosystem, depending on the plant functional group in question. Future research integrating such traits, phylogenetic comparative methods, and long-term monitoring could further elucidate the evolutionary and demographic processes underpinning these patterns. Given their sensitivity to microenvironmental conditions and their turnover-driven response, understory ferns emerge as potent indicators of fine-scale environmental change in forest ecosystems.

## Data Availability

The raw data supporting the conclusions of this article will be made available by the authors, without undue reservation.
