# Supplementary material for: The primacy of species turnover over intraspecific variation in the environmental filtering of understory ferns
Source: Front Plant Sci. 2026 Feb 25;17:1779523. doi: 10.3389/fpls.2026.1779523 (PMC12975749; doi:10.3389/fpls.2026.1779523)
Supplement: Supplementary file 1 [file DataSheet1.docx]

Supplementary Material for

The Primacy of Species Turnover over Intraspecific Variation in the Environmental Filtering of Understory Ferns

Yuhan Zhou^1^, Zhenzhen Zhang^1,2^, Heming Liu^2^, Shan Jiang^1^, Zemei Zheng^1^, Guochun Shen^1^, Xihua Wang^1^, Qingsong Yang^1*^

^1^Zhejiang Tiantong Forest Ecosystem National Observation and Research Station, School of Ecological and Environmental Sciences, East China Normal University, Shanghai, China

^2^Eastern China Conservation Centre for Wild Endangered Plant Resources, Shanghai Chenshan Botanical Garden, Shanghai, China

*** Correspondence:**Qingsong Yang
qsyang@des.ecnu.edu.cn

# Supplementary Table 1 Overview of woody plant species recorded in 121 sample plots, including family, species code, species name, importance value (*IV*), abundance (*N*) and leaf habit.

| **Family** | **Species Code** | **Species Name** | **Importance Value (%)** | **Abundance (N)** | **Leaf Habit** |
| --- | --- | --- | --- | --- | --- |
| Pentaphylacaceae | EURLOQ | *Eurya loquaiana* | 14.75 | 2696 | Evergreen |
| Lauraceae | LITELO | *Litsea elongata* | 9.26 | 1091 | Evergreen |
| Anacardiaceae | CHOAXI | *Choerospondias axillaris* | 6.15 | 55 | Deciduous |
| Theaceae | CAMFRA | *Camellia fraterna* | 5.09 | 669 | Evergreen |
| Hamamelidaceae | DISMYR | *Distylium myricoides* | 4.64 | 402 | Evergreen |
| Theaceae | SCHSUP | *Schima superba* | 4.64 | 80 | Evergreen |
| Lauraceae | MACTHU | *Machilus thunbergii* | 3.74 | 195 | Evergreen |
| Fagaceae | CYCSES | *Quercus sessilifolia* | 3.58 | 103 | Evergreen |
| Fagaceae | LITHAR | *Lithocarpus harlandii* | 3.35 | 213 | Evergreen |
| Lauraceae | NEOAUR | *Neolitsea aurata* var. *chekiangensis* | 3.29 | 332 | Evergreen |
| Fagaceae | CASFAR | *Castanopsis fargesii* | 2.98 | 75 | Evergreen |
| Pentaphylacaceae | CLEJAP | *Cleyera japonica* | 2.62 | 184 | Evergreen |
| Lauraceae | MACLEP | *Machilus leptophylla* | 2.23 | 94 | Evergreen |
| Symplocaceae | SYMANO | *Symplocos anomala* | 2.05 | 250 | Evergreen |
| Lauraceae | CINSUB | *Cinnamomum subavenium* | 2.04 | 100 | Evergreen |
| Daphniphyllaceae | DAPOLD | *Daphniphyllum oldhamii* | 1.81 | 85 | Evergreen |
| Ericaceae | RHOOVA | *Rhododendron ovatum* | 1.68 | 223 | Evergreen |
| Lauraceae | SASTZU | *Sassafras tzumu* | 1.42 | 18 | Deciduous |
| Symplocaceae | SYMSUM | *Symplocos sumuntia* | 1.40 | 102 | Evergreen |
| Pentaphylacaceae | EURRUB | *Eurya rubiginosa* var. *attenuata* | 1.38 | 154 | Evergreen |
| Fagaceae | CASCAR | *Castanopsis carlesii* | 1.35 | 40 | Evergreen |
| Symplocaceae | SYMLAN | *Symplocos lancifolia* | 1.26 | 76 | Evergreen |
| Schisandraceae | ILLLAN | *Illicium lanceolatum* | 1.26 | 85 | Evergreen |
| Symplocaceae | SYMLUC | *Symplocos lucida* | 1.18 | 84 | Evergreen |
| Oleaceae | OSMCOO | *Osmanthus cooperi* | 1.05 | 63 | Evergreen |
| Aquifoliaceae | ILEBUE | *Ilex buergeri* | 1.05 | 62 | Evergreen |
| Rosaceae | LAUPHA | *Prunus phaeosticta* | 0.87 | 65 | Evergreen |
| Fagaceae | CYCMYR | *Quercus myrsinifolia* | 0.85 | 40 | Evergreen |
| Betulaceae | CARVIM | *Carpinus viminea* | 0.78 | 18 | Deciduous |
| Rosaceae | PHOGLA | *Photinia glabra* | 0.75 | 65 | Evergreen |
| Sapindaceae | ACEPUB | *Acer pubinerve* | 0.69 | 22 | Deciduous |
| Euphorbiaceae | VERFOR | *Vernicia fordii* | 0.63 | 25 | Deciduous |
| Styracaceae | ALNFOR | *Alniphyllum fortunei* | 0.62 | 34 | Deciduous |
| Symplocaceae | SYMCOC | *Symplocos theophrastifolia* | 0.62 | 41 | Evergreen |
| Myrtaceae | SYZBUX | *Syzygium buxifolium* | 0.61 | 64 | Evergreen |
| Aquifoliaceae | ILEROT | *Ilex rotunda* | 0.61 | 26 | Evergreen |
| Pentaphylacaceae | EURMUR | *Eurya muricata* | 0.61 | 38 | Evergreen |
| Sapindaceae | SAPSAP | *Sapindus mukorossi* | 0.41 | 6 | Deciduous |
| Styracaceae | STYCON | *Styrax confusus* | 0.39 | 22 | Deciduous |
| Oleaceae | FRAINS | *Fraxinus insularis* | 0.39 | 13 | Deciduous |
| Aquifoliaceae | ILELAT | *Ilex latifolia* | 0.38 | 11 | Evergreen |
| Lauraceae | LITCOR | *Litsea coreana* var. *sinensis* | 0.34 | 16 | Evergreen |
| Lauraceae | LINRUB | *Lindera rubronervia* | 0.29 | 28 | Deciduous |
| Aquifoliaceae | ILEKEN | *Ilex kengii* | 0.28 | 11 | Evergreen |
| Ebenaceae | DIOOLE | *Diospyros oleifera* | 0.28 | 1 | Deciduous |
| Ericaceae | VACMAN | *Vaccinium mandarinorum* | 0.27 | 20 | Evergreen |
| Symplocaceae | SYMSTE | *Symplocos stellaris* | 0.26 | 13 | Evergreen |
| Fagaceae | CYCGRA | *Quercus shennongii* | 0.26 | 13 | Evergreen |
| Hamamelidaceae | LORCHI | *Loropetalum chinense* | 0.25 | 18 | Evergreen |
| Myricaceae | MYRRUB | *Morella rubra* | 0.21 | 12 | Evergreen |
| Pentaphylacaceae | TERGYM | *Ternstroemia gymnanthera* | 0.21 | 14 | Evergreen |
| Lauraceae | LITCUB | *Litsea cubeba* | 0.20 | 14 | Deciduous |
| Fagaceae | CYCGIL | *Quercus gilva* | 0.19 | 6 | Evergreen |
| Altingiaceae | LIQFOR | *Liquidambar formosana* | 0.18 | 2 | Deciduous |
| Juglandaceae | CYCPAL | *Cyclocarya paliurus* | 0.15 | 2 | Deciduous |
| Anacardiaceae | TOXSUC | *Toxicodendron succedaneum* | 0.15 | 4 | Deciduous |
| Symplocaceae | SYMBOT | *Symplocos sumuntia* | 0.14 | 4 | Evergreen |
| Fabaceae | DALHUP | *Dalbergia hupeana* | 0.14 | 3 | Deciduous |
| Fagaceae | CASSCL | *Castanopsis sclerophylla* | 0.13 | 7 | Evergreen |
| Ebenaceae | DIOJAP | *Diospyros japonica* | 0.13 | 2 | Deciduous |
| Lamiaceae | CLECYR | *Clerodendrum cyrtophyllum* | 0.12 | 12 | Deciduous |
| Cannabaceae | APHASP | *Aphananthe aspera* | 0.11 | 2 | Deciduous |
| Fagaceae | CYCGLA | *Quercus glauca* | 0.11 | 9 | Evergreen |
| Rosaceae | RHAIND | *Rhaphiolepis indica* | 0.11 | 6 | Evergreen |
| Oleaceae | OSMFRA | *Osmanthus fragrans* | 0.10 | 12 | Evergreen |
| Cannabaceae | CELVAN | *Celtis vandervoetiana* | 0.09 | 3 | Deciduous |
| Lauraceae | PHOSHE | *Phoebe sheareri* | 0.09 | 4 | Evergreen |
| Schoepfiaceae | SCHJAS | *Schoepfia jasminodora* | 0.08 | 2 | Deciduous |
| Lauraceae | LINGLA | *Lindera glauca* | 0.07 | 6 | Deciduous |
| Fagaceae | LITGLA | *Lithocarpus glaber* | 0.07 | 1 | Evergreen |
| Ericaceae | VACBRA | *Vaccinium bracteatum* | 0.06 | 3 | Evergreen |
| Rosaceae | CERDIS | *Prunus discoidea* | 0.06 | 2 | Deciduous |
| Cephalotaxaceae | CEPFOR | *Cephalotaxus fortunei* | 0.05 | 3 | Evergreen |
| Fabaceae | ALBKAL | *Albizia kalkora* | 0.04 | 1 | Deciduous |
| Cornaceae | CORCON | *Cornus controversa* | 0.03 | 1 | Deciduous |
| Sapindaceae | ACEACU | *Acer acutum* | 0.03 | 1 | Deciduous |
| Aquifoliaceae | ILECHI | *Ilex chinensis* | 0.03 | 1 | Evergreen |
| Aquifoliaceae | ILEMIC | *Ilex micrococca* | 0.03 | 1 | Deciduous |
| Ericaceae | VACTRI | *Vaccinium trichocladum* | 0.03 | 2 | Evergreen |
| Ericaceae | RHOSIM | *Rhododendron simsii* | 0.03 | 2 | Deciduous |
| Cannabaceae | CELBIO | *Celtis biondii* | 0.03 | 1 | Deciduous |
| Cannabaceae | TRECAN | *Trema cannabina* var. *dielsiana* | 0.02 | 1 | Deciduous |
| Lamiaceae | PREMIC | *Premna microphylla* | 0.02 | 1 | Deciduous |
| Euphorbiaceae | MALAPE | *Mallotus apelta* | 0.02 | 1 | Deciduous |
| Rosaceae | PHOPAR | *Photinia parvifolia* | 0.02 | 1 | Deciduous |
| Cornaceae | ALAKUR | *Alangium kurzii* | 0.02 | 1 | Deciduous |

# Supplementary Table 2 Overview of all the fern understorey species identified and the number of plots in which they were sampled (n).

| **Species Name** | **n** | **Species Name** | **n** |
| --- | --- | --- | --- |
| *Woodwardia japonica* | 113 | *Deparia dimorphophyllum* | 4 |
| *Dryopteris dehuaensis* | 111 | *Parathelypteris japonica* | 3 |
| *Arachniodes aristata* | 80 | *Athyrium otophorum* | 3 |
| *Diplazium mettenianum* | 71 | *Anisocampium sheareri* | 3 |
| *Dryopteris erythrosora* | 62 | *Athyrium iseanum* | 3 |
| *Diplopterygium glaucum* | 59 | *Pteris multifida* | 2 |
| *Arachniodes hekiana* | 42 | *Dryopteris uniformis* | 2 |
| *Arachniodes simplicior* | 41 | *Pyrrosia lingua* | 2 |
| *Dryopteris fuscipes* | 34 | *Polystichum balansae* | 2 |
| *Microlepia marginata* | 34 | *Parathelypteris chinensis* | 1 |
| *Dryopteris decipiens* | 32 | *Plagiogyria japonica* | 1 |
| *Dryopteris zhangii* | 27 | *Dryopteris pacifica* | 1 |
| *Dryopteris championii* | 26 | *Phegopteris decursive-pinnata* | 1 |
| *Lepisorus superficialis* | 25 | *Athyriaceae-sp1* | 1 |
| *Asplenium normale* | 24 | *Athyriaceae-sp2* | 1 |
| *Deparia lancea* | 17 | *Asplenium wrightii* | 1 |
| *Parathelypteris glanduligera* | 15 | *Sitobolium zeylanicum* | 1 |
| *Arachniodes speciosa* | 14 | *Pyrrosia petiolosa* | 1 |
| *Dicranopteris pedata* | 13 | *Dryopteris dickinsii* | 1 |
| *Macrothelypteris oligophlebia* | 12 | Unknown | 1 |
| *Lepisorus ovatus* | 8 |  |  |
| *Odontosoria chinensis* | 7 |  |  |
| *Coniogramme japonica* | 7 |  |  |
| *Polystichum polyblepharum* | 7 |  |  |
| *Diplazium wichurae* | 6 |  |  |

# Supplementary Figure 1 Fern community composition within the 121 plots. (A) Distribution of relative fern cover (percentage of fern cover relative to the total herbaceous layer cover) across the plots. (B) Distribution of fern species richness (number of fern species) per 10m × 10m plot.

**
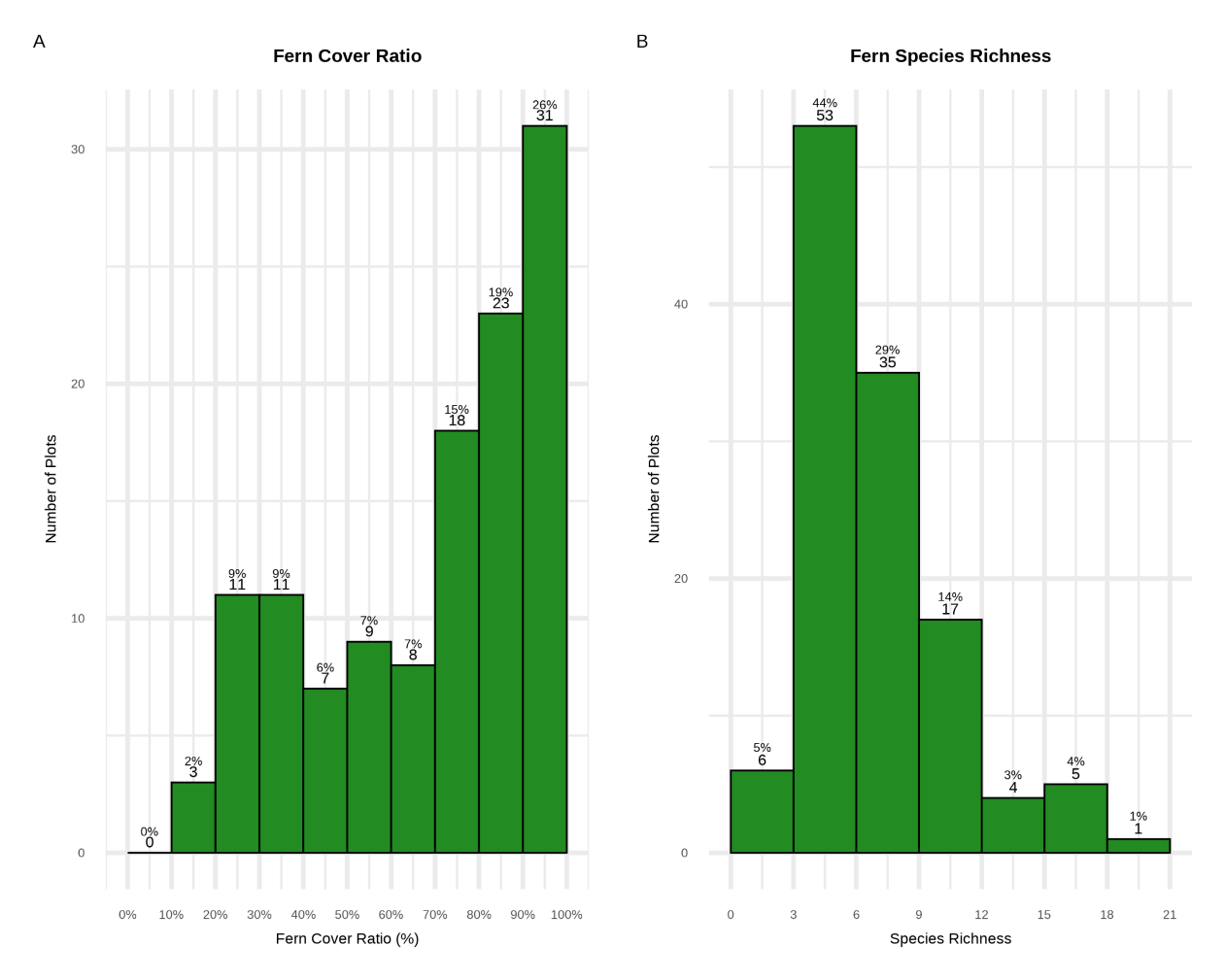
**

# Supplementary Figure 2 The Top 20 most dominant fern species, ranked by mean Importance Value (IV).

**
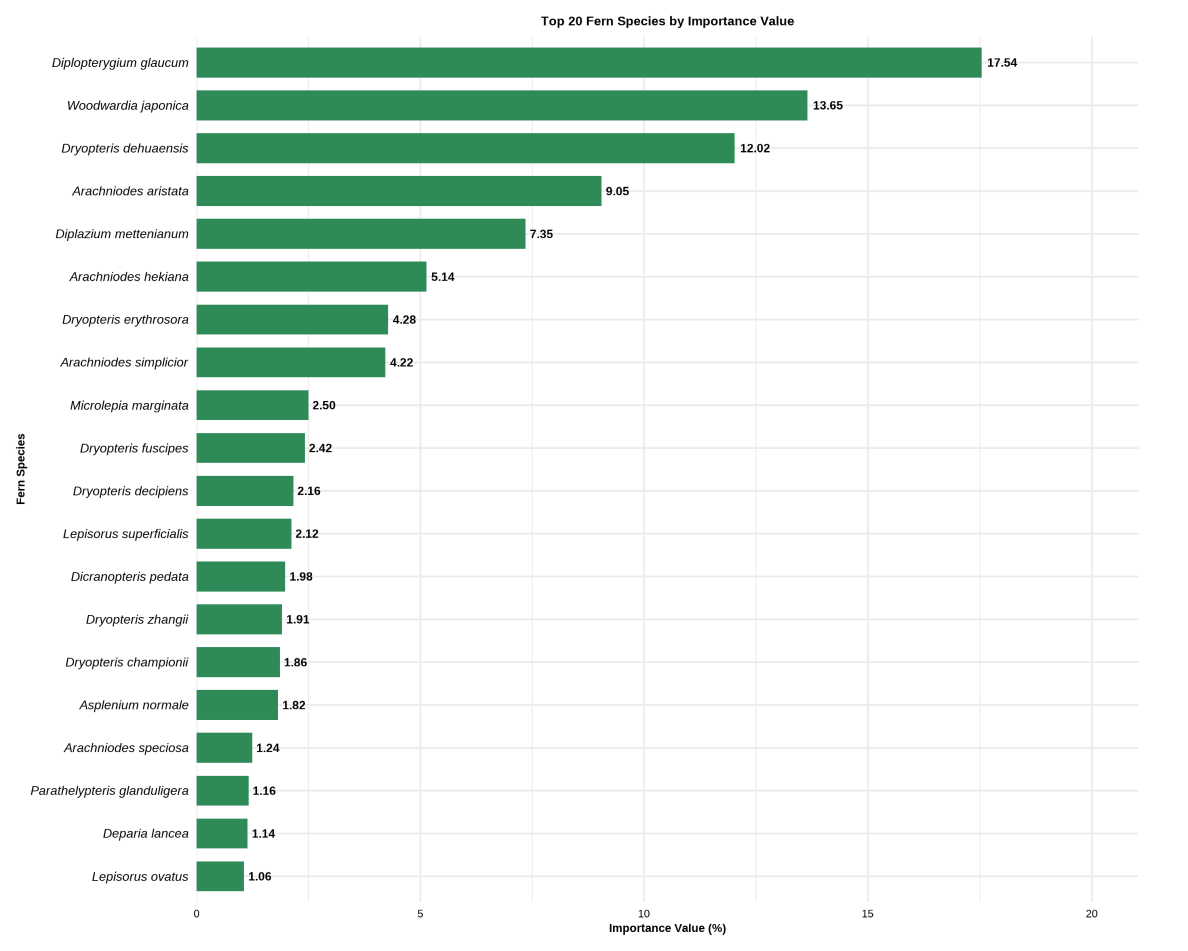
**

# Supplementary Figure 3 NMDS ordination of 121 forest sample plots based on woody plant composition. Plots are colored according to the proportion of deciduous basal area, ranging from green (evergreen-dominated) to orange (deciduous-dominated). Species scores are colored by leaf habit (blue = evergreen, red = deciduous).

**
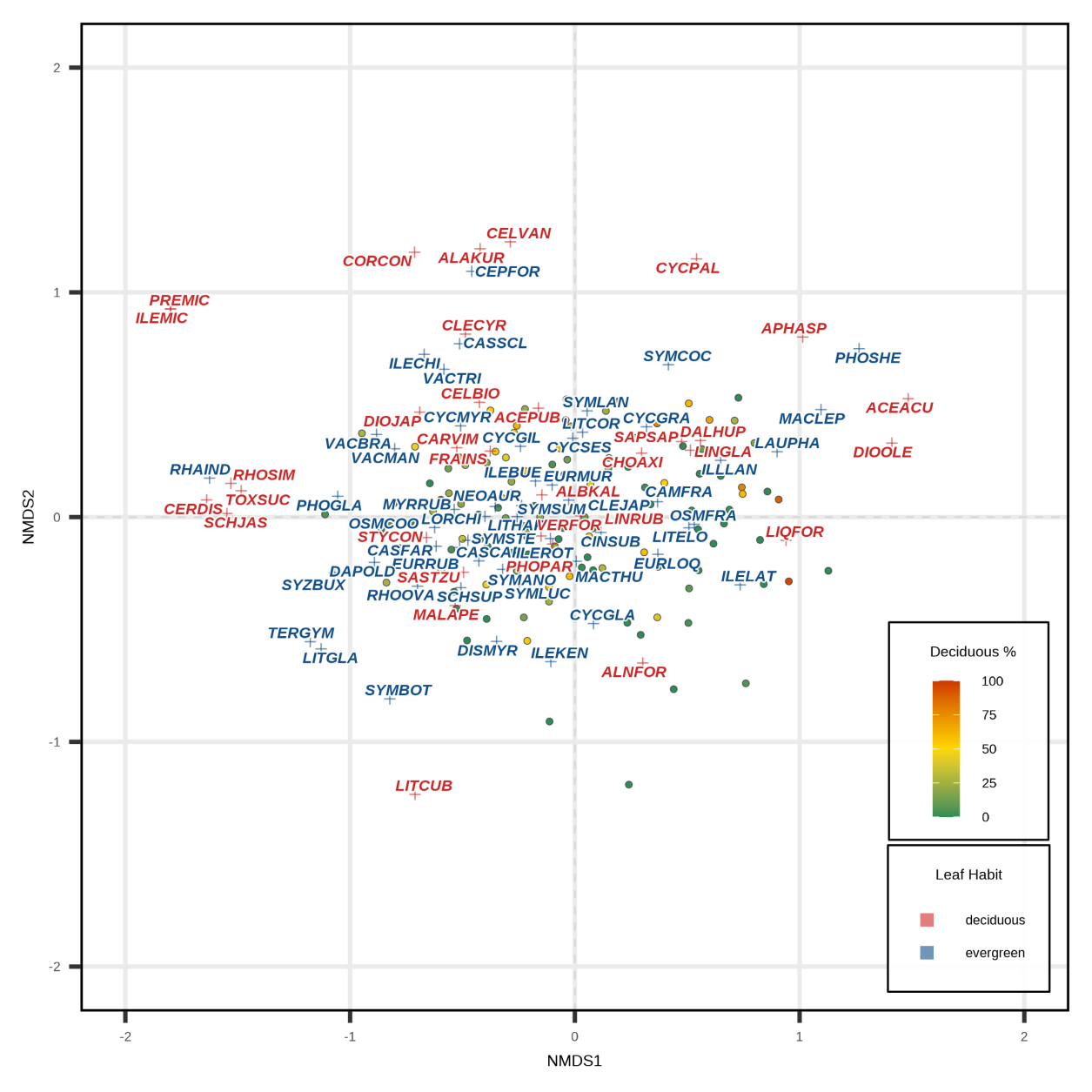
**

# Supplementary Table 3 Variance Inflation Factors (VIF) for the eight environmental predictors. Results of the VIF test for the eight predictors used in the multiple linear regression models. All values are below 5, indicating that multicollinearity was not a significant issue.

| **Predictor Variable** | **VIF** |
| --- | --- |
| PC_Topo-ele_ | 3.29 |
| PC_Topo-asp_ | 1.22 |
| PC_Soil-pH_ | 2.71 |
| PC_Soil-P_ | 2.14 |
| Density | 2.37 |
| Basal area | 1.26 |
| NMDS1 | 3.54 |
| NMDS2 | 1.89 |

# Supplementary Figure 4 Relative contributions of topographic, soil, and biotic factors to the functional traits of understory fern communities. The figure shows the results from the variance partitioning analysis based on Generalized Least Squares (GLS) models accounting for spatial autocorrelation. The values in each Venn diagram represent the percentage of variation (Nagelkerke's pseudo-R^2^) explained by each component. The non-overlapping areas of the ellipses represent the unique effects of a single factor group, while the overlapping areas represent the shared effects among factor groups. The total pseudo-R^2^ value noted below each diagram indicates the total variation explained by all three environmental factor groups combined. Note that for LDMC and Φ_PSII_, the environmental models yielded negligible explained variance (pseudo-R^2^≈0), resulting in empty plots.


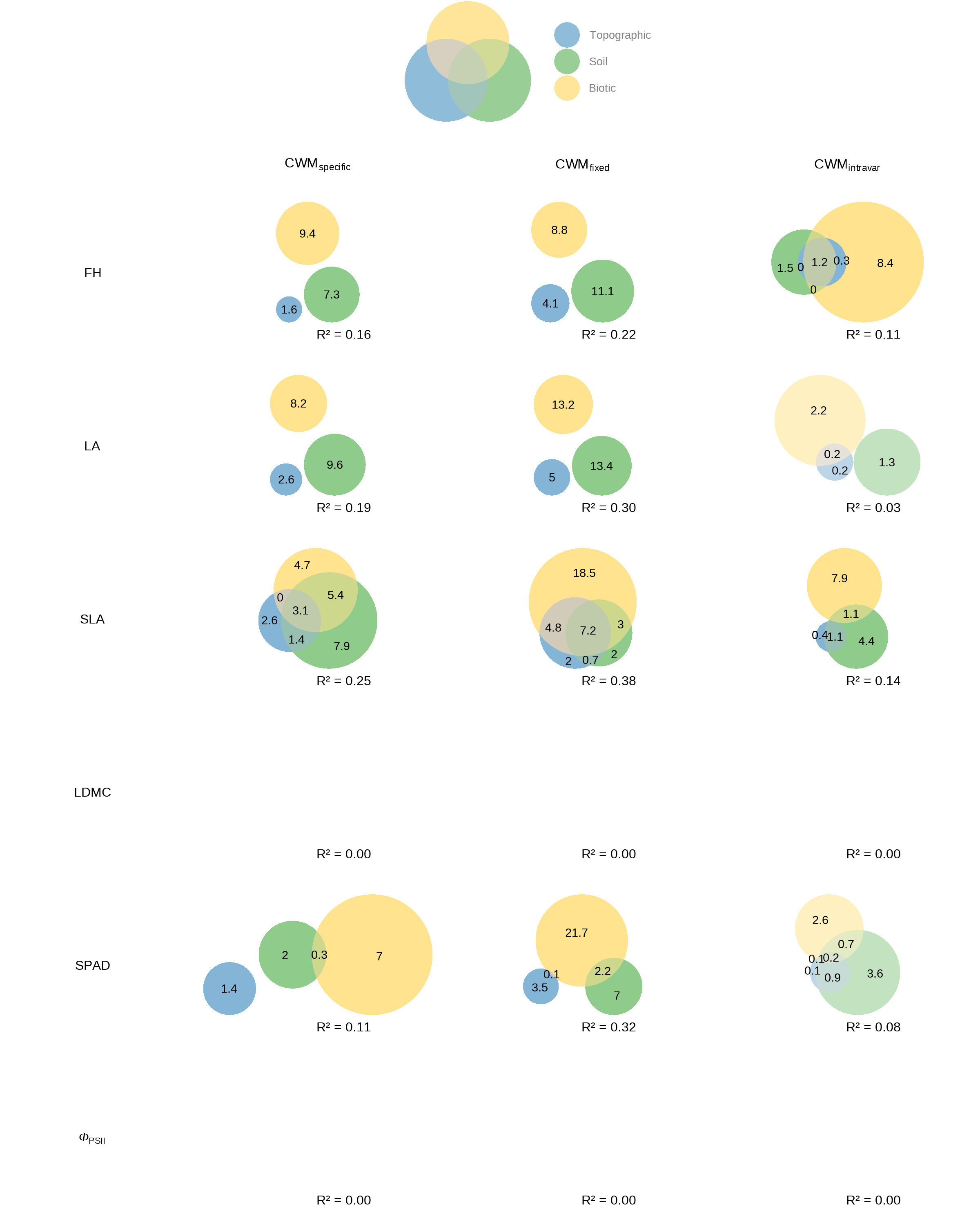


# Supplementary Figure 5 Unique effects of environmental gradients on the community-weighted means (CWM) of understory fern functional traits. Each panel displays the predicted relationship between a CWM and a single environmental predictor variable, based on a Generalized Least Squares (GLS) model that accounts for spatial autocorrelation while statistically controlling for the effects of the other seven predictors (by holding them at their mean values). Models based on CWM_specific_ are shown in green, and those based on CWM_fixed_ are in blue-green. Trend lines are shown for significant predictors, with line style indicating the level of significance: solid for *p* < 0.01 and dashed for *p* < 0.05. Non-significant relationships (*p* ≥ 0.05) are not displayed.


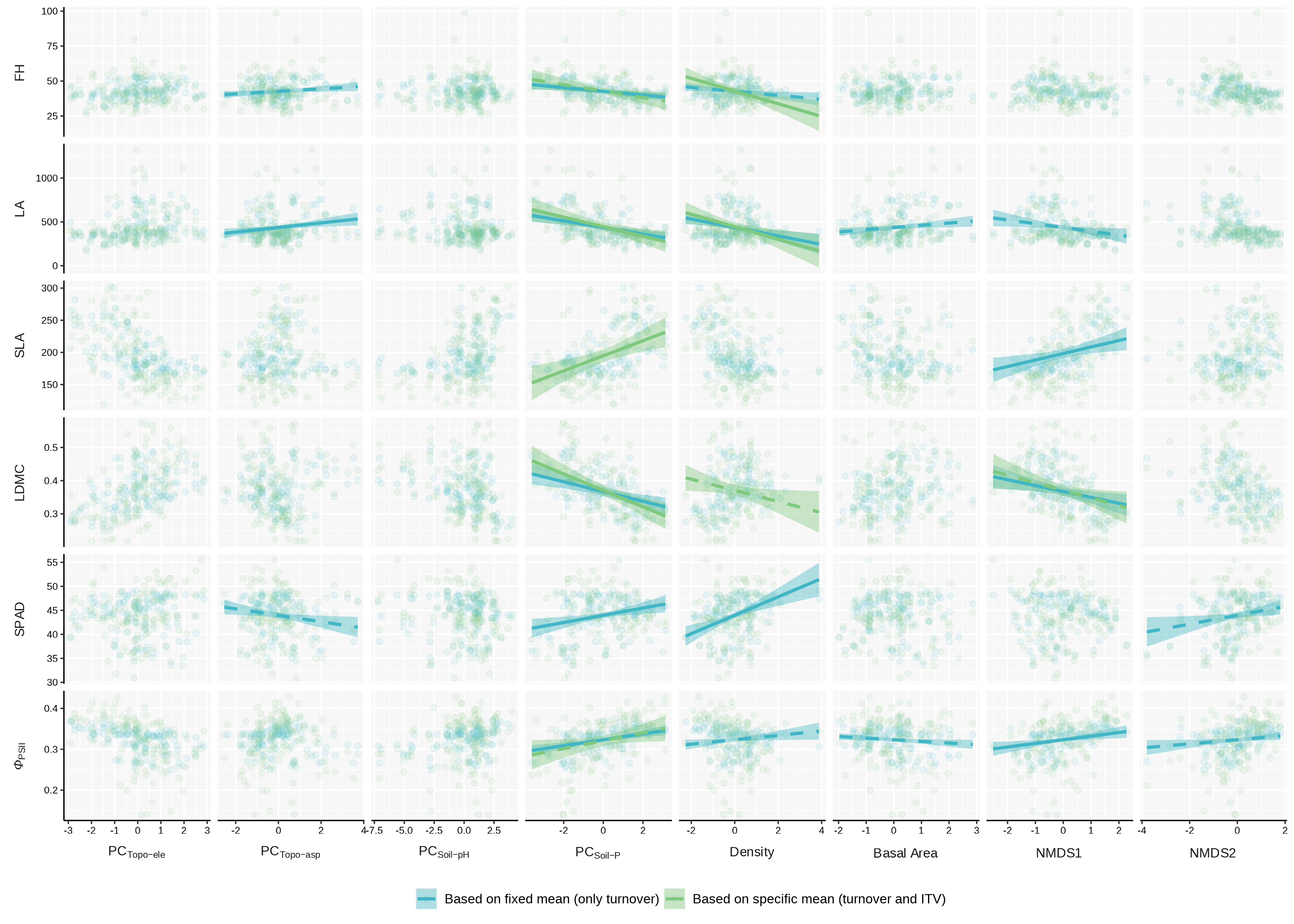


# Supplementary Figure 6 Unique effects of environmental gradients on the intraspecific variation component (CWM_intravar_) of understory fern integrated functional axes and functional traits. Each panel displays the predicted relationship between a CWM_intravar_ and a single environmental predictor variable, based on a Generalized Least Squares (GLS) model that accounts for spatial autocorrelation while statistically controls for the effects of the other seven predictors (by holding them at their mean values). Trend lines are shown for significant predictors, with line style indicating the level of significance: solid for *p* < 0.01 and dashed for *p* < 0.05. Non-significant relationships (*p* ≥ 0.05) are not displayed.

**

**

# Supplementary Table 4 Number of species exhibiting significant positive (+) or negative (−) responses to environmental predictors for each functional trait. The analysis was restricted to the 19 most abundant species to ensure sufficient data coverage across plots and statistical robustness. The statistical approach (GLS models) followed the framework used in the community-level analysis.

| **Trait** | **Factors Group** | **Positive (n)** | **Negative (n)** | **Total Responding (%)** |
| --- | --- | --- | --- | --- |
| FH | Topographic | 0 | 0 | 0% |
| FH | Soil | 0 | 2 | 11% |
| FH | Biotic | 1 | 4 | 26% |
| LA | Topographic | 1 | 0 | 5% |
| LA | Soil | 0 | 1 | 5% |
| LA | Biotic | 1 | 1 | 11% |
| SLA | Topographic | 2 | 3 | 26% |
| SLA | Soil | 2 | 0 | 11% |
| SLA | Biotic | 4 | 1 | 26% |
| LDMC | Topographic | 1 | 0 | 5% |
| LDMC | Soil | 0 | 8 | 42% |
| LDMC | Biotic | 2 | 6 | 42% |
| SPAD | Topographic | 2 | 0 | 11% |
| SPAD | Soil | 0 | 4 | 21% |
| SPAD | Biotic | 3 | 1 | 21% |
| Φ_PSII_ | Topographic | 0 | 3 | 16% |
| Φ_PSII_ | Soil | 3 | 0 | 16% |
| Φ_PSII_ | Biotic | 3 | 2 | 26% |

# Supplementary Figure 7 Species-specific trait responses to environmental drivers. Forest plots display the standardized regression coefficients (mean ± 95% confidence intervals) derived from Generalized Least Squares (GLS) models accounting for spatial autocorrelation. Results are shown for the top 2 dominant species based on importance value.

#
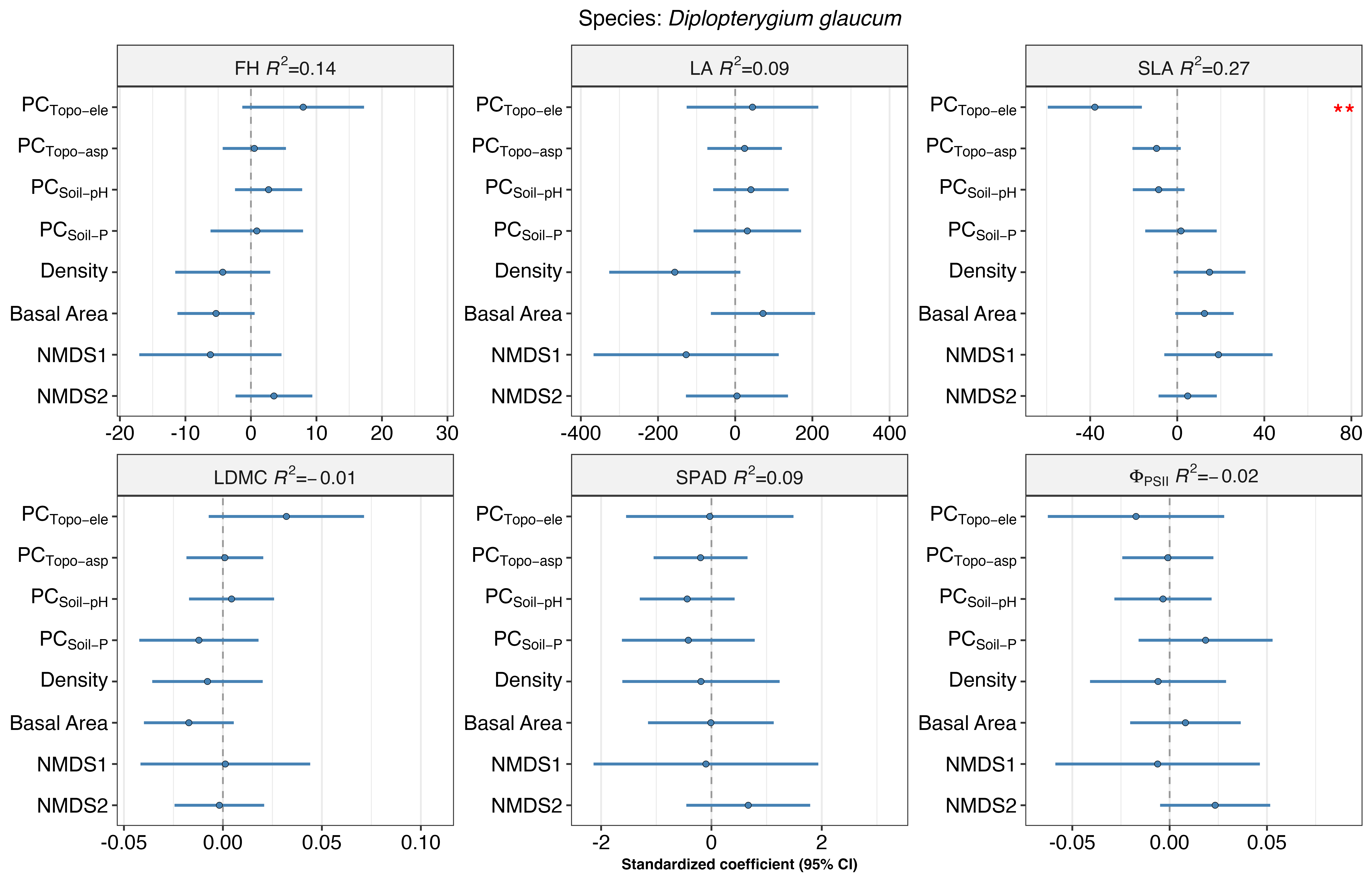


**
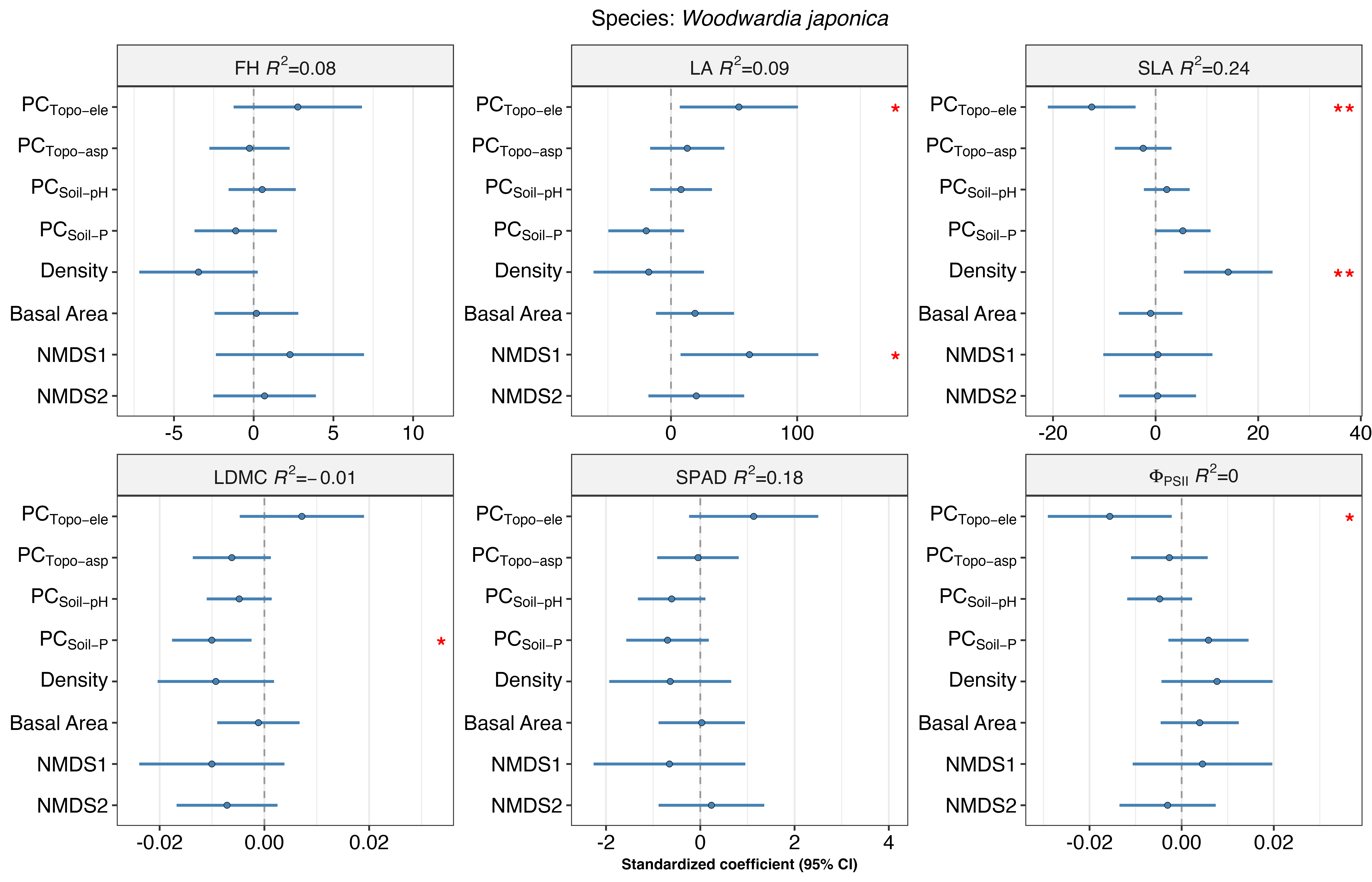
**
